# PSMOT: Online Occlusion-Aware Multi-Object Tracking Exploiting Position Sensitivity

**DOI:** 10.3390/s24041199

**Published:** 2024-02-12

**Authors:** Ranyang Zhao, Xinyan Zhang, Jianwei Zhang

**Affiliations:** 1 National Key Laboratory of Fundamental Science on Synthetic Vision, Sichuan University, Chengdu 610065, China; zry.xyan@gmail.com; 2 Independent Researcher, Chengdu 610095, China; xyanzhangzero@gmail.com

**Keywords:** multi-object tracking, anchor-based, position sensitivity, occlusion

## Abstract

Models based on joint detection and re-identification (ReID), which significantly increase the efficiency of online multi-object tracking (MOT) systems, are an evolution from separate detection and ReID models in the tracking-by-detection (TBD) paradigm. It is observed that these joint models are typically one-stage, while the two-stage models become obsolete because of their slow speed and low efficiency. However, the two-stage models have naive advantages over the one-stage anchor-based and anchor-free models in handling feature misalignment and occlusion, which suggests that the two-stage models, via meticulous design, could be on par with the state-of-the-art one-stage models. Following this intuition, we propose a robust and efficient two-stage joint model based on R–FCN, whose backbone and neck are fully convolutional, and the RoI-wise process only involves simple calculations. In the first stage, an adaptive sparse anchoring scheme is utilized to produce adequate, high-quality proposals to improve efficiency. To boost both detection and ReID, two key elements—feature aggregation and feature disentanglement—are taken into account. To improve robustness against occlusion, the position-sensitivity is exploited, first to estimate occlusion and then to direct the post-process for anti-occlusion. Finally, we link the model to a hierarchical association algorithm to form a complete MOT system called PSMOT. Compared to other cutting-edge systems, PSMOT achieves competitive performance while maintaining time efficiency.

## 1. Introduction

As one of the most critical tasks in computer vision, online multiple object tracking (MOT) aims to accurately identify and track objects of interest from real time video sequences, capturing their continuous motion trajectories. This task plays a pivotal role in applications related to advanced environmental perception and autonomous control. For instance, in autonomous driving [1], the surrounding environments are captured in real time by onboard cameras, radars and lidars, then MOT is applied to perceive objects, including vehicles, pedestrians and bicycles, providing precise tracking information for decision-level applications, such as path planning, collision avoidance and safe interaction with other traffic participants; in intelligent video surveillance [2], MOT is widely used to identify pedestrians within surveillance areas, offering a reliable means for flow estimation and swift response to potential threats or unusual activities.

In the field of MOT, tracking-by-detection (TBD) [3] stands out as the predominant paradigm, comprising three key sub-tasks. First, it detect objects from the current video frame. Second, it extracts the objects’ ReID features based on their bounding boxes. Third, it associates the detected objects with those from the previous frame, relying on cues such as the similarity of the ReID features and intersection over union (IoU). Within the TBD framework, the architecture of separate detection and embedding (SDE) [3] directly links these three sub-tasks sequentially. However, a notable drawback arises, as these tasks cannot share any computation. This limitation results in a disproportionately long processing time for the entire system, which prompts the exploration of joint detection and embedding (JDE) [3] architecture. The JDE methods integrate detection and ReID feature extraction into a unified model, mitigating the need for re-computation. However, this integration is neither a straightforward addition of an ReID branch to a detector [4] nor an expansion of the dimensions of the output coefficient map [5]. It introduces the new challenge of learning multiple tasks that may contradict each other. Different tasks exhibit sensitivity to various types of information derived from distinct Convolutional Neural Network (CNN) layers, so it is crucial to ensure that the shared feature maps encompass synthetic information and can be decomposed into task-specific features at the entry of the task branches. Therefore, feature aggregation and disentanglement emerge as the key elements in effectively solving the multi-task issue. Notably, many related works [6,7,8,9,10,11,12] have significantly enhanced the performance of the JDE MOT systems by adhering to these key elements. To maintain timeliness, these methods mainly focus on the design of one-stage joint models. In contrast, the two-stage joint models tend to become obsolete, due to slow speed and low efficiency.

In the JDE framework, the one-stage anchor-based models employ an end-to-end mechanism where the object’s bounding box regression occurs simultaneously with classification and ReID feature extraction. However, the latter two results stem from the regions of hypothetical anchors, rather than regressed regions, which leads to the problem of feature misalignment. In contrast, the two-stage models take a different approach by performing bounding box regression in the first stage. Consequently, classification and feature extraction in the second stage could be in closer proximity to the actual objects. Additionally, the one-stage anchor-free models leverage point-based mechanism to achieve accurate feature alignment. However, this approach introduces vulnerability: features extracted from a specific point of the object become highly susceptible to occlusion. Strategies for anti-occlusion are predominantly reliant on region-based methodologies and are difficult to be applied to the point-based models. In contrast, the two-stage models are all based on regions, offering proper conditions for various effective countermeasures against occlusion. Following this point of view, we initiate the design of a fundamental two-stage model based on R-FCN [13]. Despite the model’s light-weight RoI-wise process, inefficiency persists due to the dense and manual anchoring scheme in the first stage, leading to sub-optimal proposals and performance degradation in detection and ReID. To tackle this issue, we replace the original region proposal network (RPN) with a light-weight network [14], which generates high-quality proposals with sparse and adaptive anchors. To simultaneously enhance detection performance and ReID features, we incorporate the key elements, feature aggregation and feature disentanglement, into our basic model. In particular, the multi-layer feature fusion is employed for feature aggregation in the following backbone network [15] and feature disentanglement is achieved by embedding a neat set of convolutional layers on each task branch.

Originally employed in R–FCN to maintain translation-variance in deep features and in [16] to segment inter-class instances, position-sensitivity is exploited for anti-occlusion in our work for the first time. Capitalizing on the fact that different locations on a position-sensitive feature map are exclusively sensitive to corresponding parts of the objects, we leverage this inherent property to locate and exclude the occluded sub-regions. Specifically, for a given object proposal, we transform its position-sensitive classification map into a binary map. This binary representation, outputted by an adaptive mean-std threshold, indicates the visibility of each part of the object and could guide aggregation of the maps for classification, bounding box regression and ReID feature extraction, while effectively excluding interference caused by occlusion.

Finally, the proposed two-stage model is integrated with the hierarchical association algorithm in MOTDT [17], resulting in the complete system, named PSMOT. Experimental results demonstrate that PSMOT achieves outstanding performance and robustness while maintaining time efficiency. To sum up, the main contributions of our work are as follows:

- Reuses the two-stage model in multi-object tracking and leverages its inherent advantages in RoI-wise and region-based mechanisms to handle feature misalignment and provide conditions for anti-occlusion;
- The two-stage JDE model, extended from R-FCN, adopts a fully convolutional network structure that significantly reduces the computational burden in RoI-wise processing;
- Replaces the original RPN network, which relies on dense and predefined anchors, with a network based on adaptive sparse anchors, enabling the production of more high-quality proposals with fewer anchors. This replacement further improves the model’s efficiency;
- An efficient encoder–decoder network with multi-layer feature fusion is employed as the model’s backbone and additional convolutional layers are added at the entry of each task branch. Therefore, the highly informative shared features are first generated and then disentangled into effective task-specific features. This feature process effectively mitigates the conflicts between tasks and significantly improves the overall performance;
- Extends the application of position sensitivity to determine whether a specific part of an object is occluded. Leveraging this cue helps in active anti-occlusion by excluding the corresponding interference.

## 2. Related Work

### 2.1. Early Tracking-by-Detection MOT Methods

The characteristic of the TBD paradigm lies in the steps it takes to conduct object detection on each frame and associate objects between frames to establish their trajectories. Early research primarily focuses on constructing motion models that utilize motion features for tracking. For instance, ref. [18] models the position and velocity of targets, followed by Kalman filtering [19] to predict the bounding boxes of targets from the previous frames to the current frame, which are then individually subjected to IoU calculation, with the detection boxes obtained through Faster R-CNN [20] to create corresponding cost matrices. The Hungarian matching algorithm is subsequently employed to find the optimal match between tracked targets and detected objects. Although the motion models perform well in handling short-term occlusions, MOT methods relying solely on them still exhibit limitations, especially in complex scenarios.

Recent research has benefited from the powerful feature representation of CNNs and has focused on extracting and matching discriminative appearance features. For example, ref. [21] designs a feature extraction network based on GoogLeNet [22] specifically for extracting the appearance features of objects, whereas ref. [23] proposes a feature extraction network based on the feature pyramid, enhancing discriminative power through feature fusion. Motion features are often combined with appearance features; for example, ref. [24] constructs a unified cost matrix based on both IoU of bounding boxes and similarity of appearance features, and ref. [17] designs a scoring mechanism to eliminate unreliable detection results and motion predictions, then employs a hierarchical strategy to associate detected objects with tracked targets. In addition, ref. [25] utilizes recurrent neural networks (RNNs) to assess motion and appearance similarity between targets.

### 2.2. Joint Detection and Embedding (JDE) MOT Methods

The JDE MOT methods are introduced to streamline the redundant pipeline observed in the SDE approaches. Since the joint models are capable of handling detection and ReID feature extraction simultaneously, significant reduction in inference time can be achieved. However, the performance of these models suffers from issues concerning multi-task learning [26,27]. It has been discerned that feature aggregation and disentanglement are pivotal elements for enhancing the performance of multiple tasks concurrently. FairMOT [6] generates synthetic feature maps using the variant DLA-34 from [9]. Based on FairMOT’s model, RelationTrack [8] introduces a self-motivated module called GCD to separate shared features into detection-specific and ReID-specific representations and incorporates a transformer encoder with deformable attention, known as GTE, to enhance the ReID task. CSTrack [7] integrates a reciprocal network into the model from [5] to achieve feature disentanglement and embeds the scale-aware attention network into the ReID branch for feature enhancement. Swin-JDE [10] proposes an anchor-free JDE model based on Transformer architecture. In this model, the Patch-Expanding module is employed to improve the spatial information of feature maps and Einops Notation-based rearrangement is utilized to enhance the detection and tracking performance. To achieve real-time multi-object tracking, LMOT [11] introduces a simplified DLA-34 to extract detection features for the current image and generates efficient tracking features using a linear Transformer. RetinaMOT [12] extends object detection model Yolov5 [28] into a JDE model. To enhance the representative power of features, a series of retina-related convolutional modules are introduced in the backbone network.

Different from the aforementioned MOT methods, which use one-stage JDE models, PSMOT adopts an efficient two-stage model to accomplish detection and ReID feature extraction simultaneously.

### 2.3. Anti-Occlusion in MOT

Occlusion poses a significant challenge in MOT systems, manifesting in two primary issues. First, it can lead to missed detection, resulting in numerous interrupted trajectories of objects. The point-based models, such as FairMOT, are particularly vulnerable compared to the anchor-based models when faced with occlusion. Second, occlusion can corrupt the ReID features of tracked targets, which ultimately results in tracking drift. Many works [29,30,31,32,33] deal with occlusion based on regions. Typically, they partition the objects’ bounding boxes into blocks and process occlusion within each block. MOTs [4] tackle the problem by simultaneously addressing segmentation and extracting global attributes from appearance information, along with graph information. In [34], the representative power of the ReID feature is enhanced for each target through spatial and temporal attention. RelationTrack [8] adopts a deformable attention mechanism to avoid aggregating interference caused by occlusion. OUTrack [9] employs an occlusion estimation module to recognize and track occluded objects, which are missed by detection.

There are two types of occlusion in natural scenes: intra-class occlusion, where objects are obstructed by objects belonging to different classes, and inter-class occlusion, which involves overlaps between two objects from the same class. The latter is more challenging, as it requires instance-level cues for distinction. In this paper, we leverage the use of position-sensitivity [13,16] and transform it into an effective tool for addressing both inter-class and intra-class occlusion.

## 3. Our Approach

In this chapter, we present the technical details of PSMOT. We start by delving into the proposed two-stage JDE model, outlining its key modules and training process. Subsequently, we explore the entire operational flow of PSMOT, encompassing the model’s anti-occlusion inference and cooperative online association algorithm.

### 3.1. The Two-Stage JDE Model

**Figure 1:**
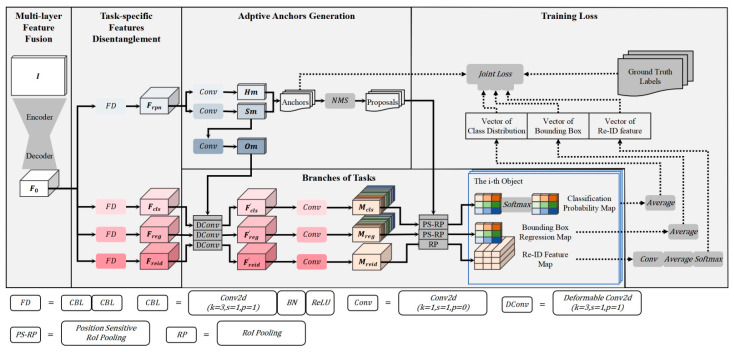
Overview of the proposed model.

The overview of our proposed model is shown in Figure 1.

The backbone network follows the encode–decode structure with a scheme of multi-layer feature fusion (see Section 3.1.1). The bottleneck network adopts the sharing of hard parameters and employs FD modules to generate task-specific feature maps (see Section 3.1.2). The anchors are automatically generated by means of adaptive anchor generation (see Section 3.1.3). Additionally, details of task branches are described in Section 3.1.4 and the joint loss function of the model is described in Section 3.1.5.

#### 3.1.1. Multi-Layer Feature Fusion

The proposed model has four tasks to complete: generation of proposals, classification, bounding box regression and the extraction of ReID features. Different tasks are sensitive to different features, which are derived from different layers of CNNs. Thus, to generate features which contain adequate information for all tasks, we employ the variant DLA-34 in FairMOT [6], which is a fully convolutional encoder–decoder network with multi-layer feature fusion, as our model’s backbone.

As shown in Figure 1, the input frame I with shape H×W×3 is fed into the backbone, which outputs the feature map F_0_ with shape $H_{0}{\;\times \;W}_{0}\times D$ where $H_{0}=H/S$, $W_{0}=W/S$, S is the output stride and D is the number of output channels. We set S to 4 to generate the feature map with relatively high resolution.

#### 3.1.2. Task-Specific Disentanglement

Through Section 3.1.1, we manage to obtain a shared feature map with strong representation, including semantic information at various levels. However, if we directly use the feature map in multi-task prediction, competitions between tasks would result in problematic or compromised convergence and cause significant decrease in the performance of each task branch. To address the issue, the shared feature map should be disentangled before being fed into each task branch.

As shown in Figure 1, we follow the idea of “Decouple Head” from Yolov8 and utilize two CBL modules to implement feature disentanglement on each branch. The essence of this process is to enhance the spatial and dimensional features exclusive to the specific task and suppress the others.

#### 3.1.3. Adaptive Sparse Anchors

The dense anchoring scheme associates every pixel in a feature map with a set of anchors with predefined scales and aspect ratios. To achieve sufficiently high recall, the total number of anchors should be large enough, which would inevitably increase the computational cost. In our model, this burden would be heavier in that the backbone outputs a high-resolution feature map. To improve efficiency, we alternatively resort to the adaptive anchoring scheme [14], which generates adaptive sparse anchors via a small fully convolutional network. This new module manages to achieve higher recall rate with fewer anchors than the original RPN-adopting dense anchoring scheme.

As shown in Figure 1, the task-specific feature map F_rpn_ undergoes two branches, location prediction branch and shape regression branch, respectively. The former branch produces a heatmap Hm indicating the probability of objects in each pixel location, and the latter generates a map of shape coefficients marked as Sm. Finally, the anchors are generated, first by selecting the locations where the probabilities of Hm are beyond a certain threshold and then choosing the most probable shape at each of the selected locations. Additionally, to keep the receptive field and semantic scope consistent with the shapes of anchors on different locations, a convolution layer is applied on the shape coefficient map to produce the offset map Om, which is used in the subsequent deformable convolution layer, employed to transform the task-specific features on task branches.

#### 3.1.4. Branches of Tasks

(1) Classification

The classification branch is designed to classify the region proposals into object categories. As shown in Figure 1, the final 1×1 convolutional layer is applied on $F_{\mathrm{cls}}^{\prime }$ to produce K^2^ groups of position-sensitive maps M_cls_. Each group contains C channels, where C is the number of categories. Given the bounding box of a proposal parameterized as (x, y, w, h), the position-sensitive RoI pooling/align is employed to produce a score map with the shape of K×K×C.

**Figure 2:**
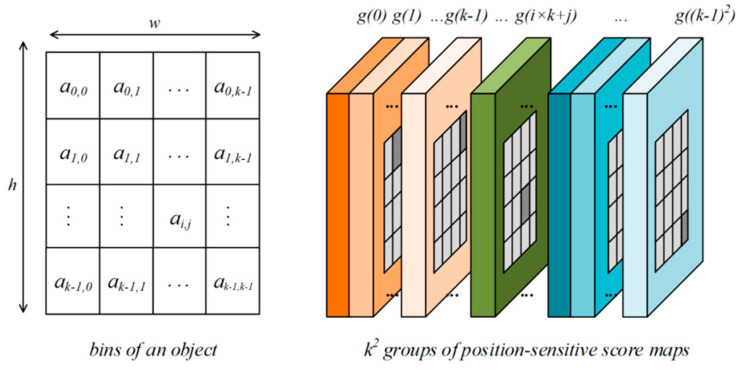
An example of the position-sensitive RoI pooling operation.

In detail, as shown in Figure 2, the bounding box is first divided into K×K bins and the value in each bin are aggregated by the vectors only from the counterpart from K^2^ groups.

For instance, the (i, j)-th bin a(i, j), which spans $X_{i}\leq x<X_{i+1}$ and $Y_{j}\leq y<Y_{j+1}$ of the bounding box, pools only at the corresponding region over the (i × k + j)-th group score map g(i × k + j), where the X_i_, X_i+1_, Y_j_ and Y_j+1_ are defined in Equation (1):

(1) Xi=i×wkXi+1=(i+1)×wkYj=j×hkYj+1=(j+1)×hk

Thereafter, the pixel-wise softmax function is applied on the score map to output the classification probability map with the same shape, as shown in Figure 1.

(2) Bounding Box Regression

The bounding box regression branch aims to align the bounding box to the correspondent object more precisely. Similar to the classification branch, the feature map $F_{\mathrm{reg}}^{\prime }$ is fed into the final convolutional layer to generate the position-sensitive maps M_reg_, for which the channels are 4K^2^, where the number 4 indicates the coefficients for a bounding box as ${(b}_{x}{,b}_{y}{,b}_{w}{,b}_{h})$, following the parameterization in [20]. Then, for each proposal, the position-sensitive RoI pooling/align is performed and produces the bounding box regression map with a shape of K×K × 4.

(3) ReID Feature Extraction

The objective of the ReID branch is to generate features that can distinguish different instances of the same class. Based on the embedding vectors used in human ReID, the branch is trained to establish an embedding space in which the vectors belonging to the same instance are close by, while those belonging to different instance are far away, according to a proper measurement. The final convolutional layer with D_t_ kernels is employed to transform the feature map and the general RoI pooling/align layer is appended to produce the ReID feature map with a shape of ${K\times K\times D}_{t}$ for each proposal. We follow the conclusion from the FairMOT that, for MOT, learning lower dimensional ReID features is more efficient, and thus set D_t_ to 64. Note that the position-sensitivity is not introduced to the ReID branch, in that the M_reid_ with position-sensitivity could be too thick to maintain balance with other tasks.

#### 3.1.5. Loss Function

The proposed network is optimized in an end-to-end fashion employing task-independent uncertainty loss [27] to balance tasks automatically. The joint loss function is formed in Equation (2), where w_i_ is the uncertainty weight for the loss of each task and can be learned as a parameter. Different from linear summation of losses, this automatic balancing method breaks the strong restriction on loss weights, so the result can be closer to the optimal value.

(2) $$L_{\mathrm{joint}}=\frac{1}{2}(\frac{L_{\mathrm{GA}}}{e^{w1}}+\frac{L_{\alpha }}{e^{w2}}+\frac{L_{\beta }}{e^{w3}}+\frac{L_{\gamma }}{e^{w4}}{+w}_{1}{+w}_{2}{+w}_{3}{+w}_{4})$$

The L_GA_ indicates the total loss of the module in Section 3.1.3, which is a linear combination of the loss of location prediction L_loc_ and the loss of shape regression L_shape,_ as shown in Equation (3):

(3) $$L_{\mathrm{GA}}{=\omega }_{1}{\;\times \;L}_{\mathrm{loc}}{\;+\;\omega }_{2}{\;\times \;L}_{\mathrm{shape}}$$

where L_loc_ is optimized by focal loss and L_shape_ is optimized by a variant of bounded IoU loss formed as Equation (4):

(4) $$L_{\mathrm{shape}}=\mathrm{SmoothL}1[1-\min(\frac{w}{w^{*}},\frac{w^{*}}{w})]+\mathrm{SmoothL}1[1-\min(\frac{h}{h^{*}},\frac{h^{*}}{h})]$$

where w and h represent predicted width and height of anchors and * represents the ground truth.

During training, the classification probability map for each proposal is additionally averaged to yield a vector with C channels, then the loss for classification branch $L_{\alpha }$ is formulated as Equation (5), where N represents the number of objects in the a frame, i refers to the i-th object and $p_{i}^{*}$ is the ground truth label of the object’s categories ($p_{i}^{*}=\bar{0}$ signifies the background).

(5) $$L_{\alpha }=\frac{1}{N}\times \sum_{i=0}^{N-1}{\mathrm{CrossEntropy}(p}_{i}{,p}_{i}^{*})$$

The bounding box regression map for each proposal is also averaged to yield a 4-d vector, and the loss for bounding box regression branch $L_{\beta }$ is calculated as in Equation (6):

(6) $$L_{\beta }=\frac{1}{N}\times \sum_{i=0}^{N-1}{[p}_{i}^{*}>0]\times {\mathrm{SmoothL}1(b}_{i}{,b}_{i}^{*})$$

where $b_{i}$ is the i-th estimated bounding box, $b_{i}^{*}$ is the corresponding ground truth vector and $\left[p_{i}^{*}>0\right]$ is an indicator which equals 1 if the argument is true and 0 otherwise.

As for the ReID branch, we train it as a classification task. As shown in Figure 1, during training, the ReID feature map of each proposal undergoes additional series of functions and is converted to a vector of a large number of categories; the objects of the same identity in the training set are treated as one class. Thus, after training, the ReID features learn to discriminate different instances. We denote the class distribution vector of a proposal as c_i_ and its corresponding one-shot representation of ground truth label as $c_{i}^{*}$, and we compute the ReID loss Lγ as Equation (7):

(7) $$L_{\gamma }=\frac{1}{\mathrm{MN}}\sum_{i-0}^{N-1}\sum_{j=0}^{M-1}{[p}_{i}^{*}>0]\times c_{i}^{*}\left[j\right]\times {\log(c}_{i}\left[j]\right)$$

where N represents the number of objects and M is the number of identity classes. 

Moreover, to take full advantage of the high-quality proposals generated by Section 3.1.3, we set a relatively high positive/negative threshold and use fewer samples during training.

### 3.2. Online Tracking

**Figure 3:**
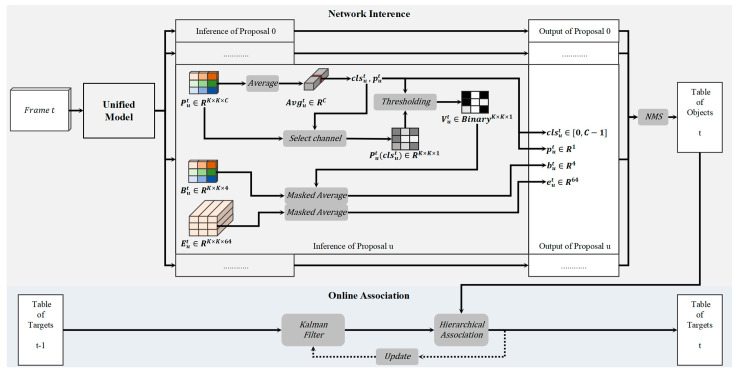
Overview of the Online Tracking Process.

The overview of our online tracking is shown in Figure 3. The whole process can be divided into network inference and online association.

#### 3.2.1. Anti-Occlusion Inference

The anchors from Section 3.1.3 indicate the regions where objects of interest are likely to be present. To eliminate duplicate anchors, the NMS function is performed on the anchors to produce a number of region proposals. We denote the classification probability map, bounding box regression map and ReID feature map of a proposal u in frame t as $P_{u}^{t}(\in R^{K\times K\times C})$, $B_{u}^{t}(\in R^{K\times K\times 4})$, $E_{u}^{t}(\in R^{K\times K\times 64})$, respectively.

We first average the vectors in K×K bins of $P_{u}^{t}$, as shown in Equation (8):

(8) $${\mathrm{Avg}}_{u}^{t}=\frac{1}{K^{2}}\times \sum_{i=0}^{K-1}\sum_{j=0}^{K-1}P_{u}^{t}\left(i,j\right)$$

Along the C channels of ${\mathrm{Avg}}_{u}^{t}$, the channel which contains the maximum value is determined as the proposal’s category, as shown in Equation (9):

(9) $${\mathrm{cls}}_{u}^{t}{=\mathrm{argmax}}_{0}^{C-1}{[\mathrm{Avg}}_{u}^{t}\left(k)\right]$$

The maximum value is formulated as Equation (10):

(10) $${\mathrm{mp}}_{u}^{t}{=\mathrm{MAX}}_{0}^{C-1}{[\mathrm{Avg}}_{u}^{t}\left(k)\right]$$

Then, we extract the ${\mathrm{cls}}_{u}^{t}$-th map from $p_{u}^{t}$ and transform it to the visibility map $V_{u}^{t}$ by binarization based on $p_{u}^{t}$ and the RMS $\sigma_{u}^{t}$, as shown in Equation (11):

(11) $$V_{u}^{t}(i,j)=\begin{array}{l} 1\;P_{u}^{t}{(i,j,\mathrm{cls}}_{u}^{t})\in {[\mathrm{mp}}_{u}^{t}-\sigma_{u}^{t}{,\mathrm{mp}}_{u}^{t}+\sigma_{u}^{t}]\\ 0\;\mathrm{else} \end{array}$$

$V_{u}^{t}(i,j)=1$ indicates that, at position (i, j), an object of class ${\mathrm{cls}}_{u}^{t}$ appears, otherwise there could be either occlusion or background. To avoid taking in irrelevant cues, we only average the values in the positions where $V_{u}^{t}(i,j)=1$ for the ${\mathrm{cls}}_{u}^{t}$-th channel of $P_{u}^{t}$, $B_{u}^{t}$ and $E_{u}^{t}$. Thus, the probability of the category ${\mathrm{cls}}_{u}^{t}$ is calculated as in Equation (12):

(12) $$p_{u}^{t}=\frac{1}{n}\sum_{i=0}^{K-1}\sum_{j=0}^{K-1}{[V}_{u}^{t}\left(i,j\right)\times P_{u}^{t}{(i,j,\mathrm{cls}}_{u}^{t})]$$

Thus, the bonding box regression vector of the proposal is shown in Equation (13):

(13) $$b_{u}^{t}=\frac{1}{n}\sum_{i=0}^{K-1}\sum_{j=0}^{K-1}{[V}_{u}^{t}\left(i,j\right)\times B_{u}^{t}\left(i,j)\right]$$

The ReID feature vector of the proposal is calculated as Equation (14):

(14) $$e_{u}^{t}=\frac{1}{n}\sum_{i=0}^{K-1}\sum_{j=0}^{K-1}{[V}_{u}^{t}\left(i,j\right)\times E_{u}^{t}\left(i,j)\right]$$

where n is the total number of positions where $V_{u}^{t}(i,j)=1$.

Along with $b_{u}^{t}$, the bounding box of the corresponding proposal u is rectified. Then the NMS function is performed on all of the proposals to generate a certain number of detection candidates, which are input to the next section.

#### 3.2.2. Online Association

To further improve the stability of our MOT system, we follow the online association strategy from MOTDT [17], which utilizes reliable detection results to prevent tracking drift in the long term, and predictions of previous tracks to avoid missed or false detection caused by occlusions. The strategy originally adopts the Euclidean distance function to evaluates the similarity of ReID feature vectors between the detection candidates and the targets; instead, we utilize the cosine distance in our work. In addition, we use the linear blending function to update the ReID feature vectors of targets when they have been successfully associated with the detection candidates; for a matched pair <${\mathrm{Target}}_{v}^{t-1}$, ${\mathrm{Detection}}_{u}^{t}$>, the ReID feature vector $\varepsilon_{v}^{t}$ of ${\mathrm{Target}}_{v}^{t}$ is updated as Equation (15):

(15) $$\varepsilon_{v}^{t}=(1-\alpha )\times \varepsilon_{v}^{t}+\alpha \times e_{u}^{t}$$

#### 3.2.3. Anti-Occlusion Tracking

Here we explain how tracking drifts are occur. From the beginning, an object is partially occluded but can still be detected. However, at this moment, the object’s bounding box contains not only the object itself but also the occlusion. Then, the ReID feature vectors in the bounding box are extracted and aggregated, bringing in the interference of occlusion. Being partially occluded, the object is successfully matched with the correctly tracked target. Then, the ReID feature of the object is updated to the tracked target as Equation (15); the ReID feature of the tracked target is contaminated by occlusion. Thereafter, the object’s ReID feature is always matched with a contaminated feature of the tracked target, leading finally to tracking drift.

In our work, the proposed MOT system is armed with the capability of anti-occlusion by position sensitivity, which encodes information on position into the K×K bins in $P_{u}^{t}{[\mathrm{cls}}_{u}^{t}]$ and $B_{u}^{t}$. For $P_{u}^{t}{[\mathrm{cls}}_{u}^{t}]$; each bin responds with high confidence only to the corresponding part of an object. Therefore, we can directly use the strength of the response to determine whether the correspondent area is obstructed by occlusions, which might be either inter-class or intra-class. To exclude the irrelevant information from the occluded parts, we merely aggregate the vectors on the bins that are not occluded. Therefore, the tracking drifts are effectively resolved by ensuring the object’s and its matched target’s ReID feature are uncontaminated.

## 4. Experiments

In this chapter, we apply our PSMOT to online multi-pedestrian tracking and evaluate this via various corresponding public datasets.

### 4.1. Datasets

In order to train the proposed unified model, we combine eight public datasets, ETH [35], CityPerson [36], WiderPerson-traffic [37], CalTech [38], MOT16 [39], CUHK-SYSU [40], PRW [41] and TAO-person [42], to create a large-scale training set for pedestrian detection and ReID. The ETH, CityPerson and WiderPerson-traffic datasets are utilized to train the classification and bounding box regression branches, because they only offer bounding box annotations. Together, the remaining datasets offering identification and box annotations are used to train all the task branches. We assess our method using the MOT17 [39] and MOT20 [43] testing sets after training.

### 4.2. Metrics

We evaluate the performance of the proposed unified model in three areas. First, the detection accuracy [44] (DetA) and localization accuracy [44] (LocA) are used to assess the detection performance. Second, the association accuracy [44] (AssA) evaluates the discriminability of the ReID features. Thirdly, the number of switches in the targets’ identification [45] (IDs) and the fragments of the targets’ trajectories [45] (Frag) are used to assess the quality of their predicted trajectories. Additionally, two comprehensive metrics are utilized to evaluate overall performance: the MOTA [46] and IDF1 [47]. The FPS is employed to measure the processing speed, whose reciprocal is referred to as the inference time in seconds.

### 4.3. Implementation

The modified version of the DLA-34, whose parameters are pre-trained on the COCO dataset [48], is employed as the backbone of our unified model. For online pedestrian tracking, the number of categories is set as 1. Besides, in default, the dimension of the ReID features is 64 and the size of the spatial grid K is 5. The hyper-parameters of the module in Section 3.1.3 follow the parameterization in [14], with $\sigma_{1}=0.2$, $\sigma_{2}=0.2$, $\omega_{1}=1$ and $\omega_{2}=0.1$, and the number of the proposals is manually limited to 500. The standard Adam optimizer [49] is employed to implement the data fitting. Specifically, the number of training epochs is 30 and the learning rate is initialized as 0.02 and dynamically decreased by 10% at the 15th and 25th epoch. The batch size is set as 10. Besides, to further improve the performance of our trackers, standard training schemes, such as the online hard example mining (OHEM) [50] and data augmentation techniques [51], are employed during their training. The training takes about 43 h on two RTX 2080Ti GPUs.

### 4.4. Ablastion Studies

#### 4.4.1. Multi-Layer Feature Fusion

In this section we examine the efficacy of multi-layer feature fusion. Two models with other distinct backbones are produced as the control group, in addition to the variant DLA-34 in the proposed model. These models are the ResNet-34 [52] and the FPN-34 [53], which is the ResNet-34 with the feature pyramid structure. All models have a stride of 4, and the ResNet-34 requires the integration of three extra up-sampling operations in order to maintain its stride.

The results are shown in Table 1. By comparing the FPN-34 with the ResNet-34, it is evident that the AssA, DetA and LocA improve significantly. We credit these advancements to the usage of multi-layer feature fusion in the FPN-34. Furthermore, the DLA-34 achieves even greater results with its encoder–decoder layout and additional levels of feature fusion. In particular, there is a 4.4%, 6.3% and 5.1% increase in AssA, DetA and LocA compared with the ResNet-34, respectively. A strong foundation for tracking is provided by high-precision detection and discriminative ReID features, which inevitably lead to better tracking performance. The table shows a significant increase in MOTA and IDF1 and a decrease in IDs and Frag. Consequently, the results imply that our feature aggregation scheme effectively mitigates the conflicts between tasks and significantly improves the overall performance.

#### 4.4.2. Feature Disentanglement

As for the evaluation of the proposed module for feature disentanglement, we compare it with the general method, which simply transforms features by the combination of 3×3 convolution and 1×1 convolution on each task branch.

The results are shown in Table 2. Our solution achieves a noticeable improvement in performance by substituting the dual CBL layers at the entrance of each task branch for the general module. The AssA, DetA and LocA increase by 2.6%, 2.4% and 2.5%, respectively, showing that our feature disentanglement method helps to resolve conflicts better among tasks.

#### 4.4.3. Generation of Adaptive Anchors

In this section, we compare two versions of PSMOT: PSMOT with RPN based on adaptive anchors generation and PSMOT with vanilla RPN. We set the maximum number of proposals from 300 to 1000, respectively, and fix the IoU threshold to 0.6 in order to define the positive and negative samples.

The results are shown in Table 3. As for the vanilla RPN, the MOTA of the MOT system improves by 1.4% when the maximum number of proposals increases from 300 to 1000, while the FPS noticeably decreases from 14.3 Hz to 5.1 Hz. Nevertheless, the proposed PSMOT obtains substantially greater performance in a shorter amount of operating time when the generation of adaptive anchors is applied to the RPN: the FPS increases from 14.3 Hz to 22.4 Hz, the MOTA increases from 72.1% to 73.2%, and the IDF1 increases from 72.5% to 74.4%. We attribute the gains in overall performance to the higher yield rate of high-quality proposals produced by the generation of adaptive anchors and the gains in FPS to the sparse anchors scheme. Furthermore, it is evident that the system performance will continue to improve as we loosen the limit on the number of proposals in order to introduce more high-quality proposals, but the computation time will also increase.

#### 4.4.4. Position Sensitivity

The position sensitivity in PSMOT is essential for handling occlusions. Therefore, it is essential to assess this attribute’s efficiency. The results are shown in Table 4. It should be noted that, when K = 1, the position-sensitive pooling/align is deteriorated to the global pooling/align and the position-sensitive feature maps are relegated; as a result, the anti-occlusion is removed from the PSMOT.

With the help of position sensitivity, the performance improves significantly, with only a small increase in running time. Specifically, the quality of the tracked trajectories improves significantly: the IDs reduce from 535 to 252 and the Frag from 878 to 504, indicating a successful suppression of the tracking drift issue.

We also look into the impact of the grid dimensions K. As the number of the grid dimensions increases, the proposals’ granularity becomes finer, which makes it easier to detect and associate the obstructed objects. However, the processing time also increases. When K = 9, PSMOT has almost lost its timeliness, but the performance gains become noticeably slow. As the grid dimensions rise, the position-sensitive maps become thicker, making convergence more challenging.

#### 4.4.5. Association Scheme

In this section, we evaluate and analyze the impact of the adopted association scheme on the performance of PSMOT.

**ReID:** the similarity between the detected objects and the tracking targets is based on ReID features. The Hungarian algorithm is adopted to finally decide which target a certain object is assigned to.

**ReID + IoU and Kalman:** for each tracking target, the Kalman filter is adopted to predict its bounding box in the current frame. The similarity between the detected objects and the tracking targets is, additionally, based on the IoU of bounding boxes.

**ReID + IoU and Kalman + Hierarchy:** the predicted targets and the detected objects in the current frame are all considered as candidates. The hierarchy step includes selection of the candidates with a high confidence score, calculation of similarity between the candidates and the tracking targets based on ReID features and bounding boxes’ IoU, and final assignment using Hungarian algorithm. This combination constitutes the association algorithm adopted in PSMOT.

The results are shown in Table 5. Even if we only use ReID features for association, our method still exhibits good performance. The addition of motion prediction and IoU matching together contribute 1.3% and 1.0% gains to MOTA and IDF1, respectively, and reduce the number of IDs and Frag at the same time. Furthermore, utilizing a hierarchical matching and assigning scheme further boosts IDF1 by 2.2% and reduces IDs and Frag by 50 and 57, making the tracking process more sustainable. On the other hand, due to the growing number of candidates for matching and assigning, the operating speed drops from 19.0 FPS to 16.6 FPS.

### 4.5. Comparisons with State-of-the-Art MOT Methods

In this part, we compare the performance of PSMOT with the preceding SOTA online MOT trackers on the test sets of MOT17 and MOT20. In order to evaluate our approach more thoroughly, we prepare three versions of PSMOT: PSMOT-Fast, which focuses on timeliness; PSMOT-Balance, which focuses on balance between performance and timeliness; and PSMOT-Pro, which focuses on performance. The detailed configurations are shown in Table 6.

#### 4.5.1. Comparisons with Typical Methods

First, we select two representative methods for comparative analysis, which employ technical principles similar to PSMOT. These methods are FairMOT and RelationTrack, respectively. The results are shown in Table 7.

**PSMOT vs. FairMOT:** PSMOT and FairMOT achieve feature aggregation through the DLA-34 network. However, FairMOT applies linear convolutional operations to the shared feature map when passed to each task branch, while PSMOT employs non-linear convolutional operations, as mentioned in Section 3.1.2 and Section 4.4.2. Besides, FairMOT performs classification, bounding box regression and ReID feature extraction based on points, while PSMOT adopts a two-stage approach: in the first stage, it generates region proposals based on points, and in the second stage it performs classification, bounding box regression and ReID feature extraction within the proposals’ areas and utilizes position sensitivity to exclude the occluded parts. As shown in the first row and the fourth row of the table, with slight increase in parameter size (24.4 M vs. 24.8 M) and inference time (25.9 FPS vs. 20.0 FPS), PSMOT demonstrates a significant advantage in performance when compared with FairMOT.

**PSMOT vs. RelationTrack:** RelationTrack also employs the DLA-34 network to achieve feature aggregation and utilizes the Global Context Disentangling (GCD) module to decouple the shared feature map into the detection-specific and ReID-specific feature maps. However, it does not consider the conflicts between classification and localization within the detection task and only processes the detection-specific feature map with linear convolutions on the two sub-task branches. In contrast, PSMOT directly employs non-linear convolutions on the task branches to disentangle the shared feature map into proposal-specific, classification-specific, localization-specific and ReID-specific feature maps, respectively, which further alleviates the conflicts among all tasks. As shown in the table, the PSMOT series outperforms RelationTrack in terms of MOTA, LocA and DetA, with more parameters (24.8 M, 24.9 M, 25.0 M vs. 22.7 M). Additionally, RelationTrack employs the Guided Transformer Encoder (GTE) module to enhance the ReID feature map by a global self-attention mechanism, while PSMOT generates visibility maps for each proposal by position sensitivity and utilizes these to exclude the occluded parts. From the table, we can see that PSMOT-Fast performs slightly worse than RelationTrack in terms of tracking-related metrics, such as IDF1, AssA, IDs and Frag. As the region of proposals in PSMOT becomes more finely divided, the tracking performance gradually approaches that of RelationTrack. As shown in the last row of the table, PSMOT-Pro, which divides each proposal into 7 × 7 grids, exhibits a tracking performance superior to that of RelationTrack.

#### 4.5.2. Comparisons with Methods for MOT Benchmarks

Table 8 demonstrates that, in spite of its sluggish operating speed, PSMOT-Pro has outperformed its compared counterparts by significant margins in terms of the performance-related metrics. Meanwhile, PSMOT-Balance and PSMOT-Fast, when compared with the MOT methods of the one-stage JDE models, provide exceptional performance, while maintaining timeliness. In particular, PSMOT-Balance performs better than FairMOT, CSTrack, and RelationTrack by 3.5%, 3.2%, and 1.1% in the IDF1 metric at a running speed of 16.6 FPS and produces low IDs and Frags in MOT17. Even in MOT20, where the scenes are more crowded and intricate, PSMOT-Balance still surpasses them by a large margin. Furthermore, PSMOT-Fast performs better than FairMOT in MOT17 and MOT20, matching FairMOT’s speed in MOT20 and reaching nearly real-time speed in MOT17.

### 4.6. Visualization

#### 4.6.1. Visualization of the Visibility Map

**Figure 4:**
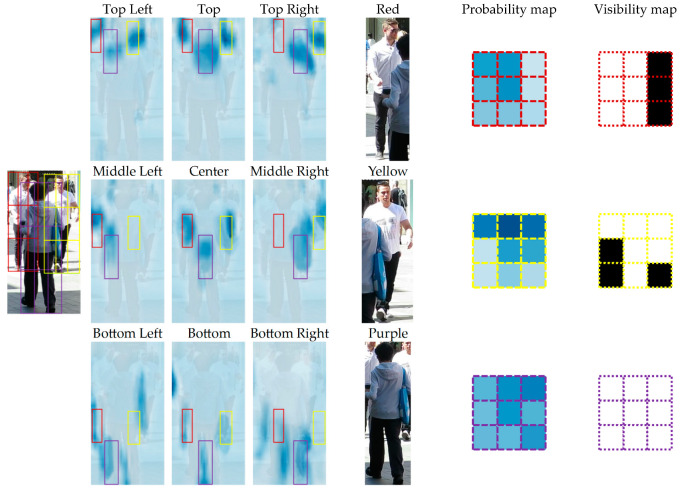
Example of the generation of targets’ visibility maps. Columns 2 to 4: the 3×3 position-sensitive maps of classification probability fused with the raw frame and the process of the position-sensitive pooling/align. Columns 6 to 7: the assembled classification probability maps and the visibility maps for the three people in this case.

The generation of visibility maps based on position sensitivity is shown visually in Figure 4. Note that each of the 3×3 bins is sensitive to a different part of the human body. For example, the top-left bin is sensitive to the left shoulder while the top-middle bin is sensitive to the head and neck.

Because of the overlap of different parts, the value in each bin of the probability map can be directly used to determine if the corresponding part is obscured by another instance of the same class. Next, we use mean-deviation threshold to convert the probability map into a binary map, which explicitly suggests the visibility of distinct parts.

We can see in the figure that the man in the purple box has blocked the bottom-left and bottom-right parts of the men in the yellow and red boxes, respectively. Their visibility maps clearly illustrate how they are obscured. Consequently, by filtering out the occluded parts, the aggregated results are more dependable than those aggregated via global averaging.

#### 4.6.2. Visualization of Detection and Tracking of Occluded Targets

**Figure 5:**
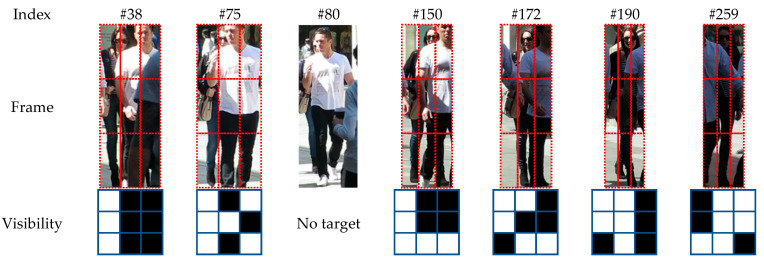
Example of detecting and tracking of a frequently occluded target. The visibility map shows the visible parts of the lady behind the man in white.

Since the region proposals are broken down into bins, as Figure 5 illustrates, our approach has an advantage when it comes to identifying the obstructed parts. As shown in the figure, even though the lady’s view is blocked by the man in front, her exposed features could still yield a clean ReID feature, thus avoiding contamination of the recorded ReID feature in the tracking pool.

**Figure 6:**
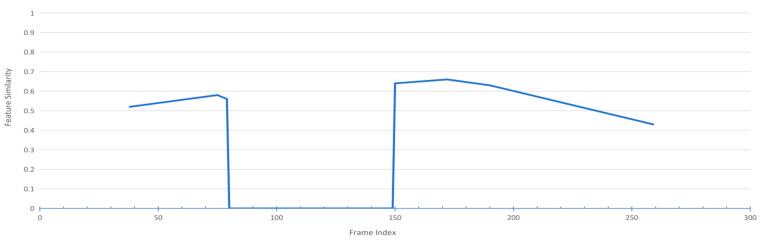
The last row quantifies the similarity between the lady’s ReID feature in each frame and that stored in the tracking pool.

Figure 6 displays the variation in similarity between the lady’s ReID feature in each frame and the corresponding tracking pool’s feature. The lady vanishes at frame 80 and her tracks are interrupted for several frames. When she reappears at frame 150, her current ReID feature still maintains high similarity with her tracking pool’s feature.

#### 4.6.3. Visualization of Online Tracking

**Figure 7:**
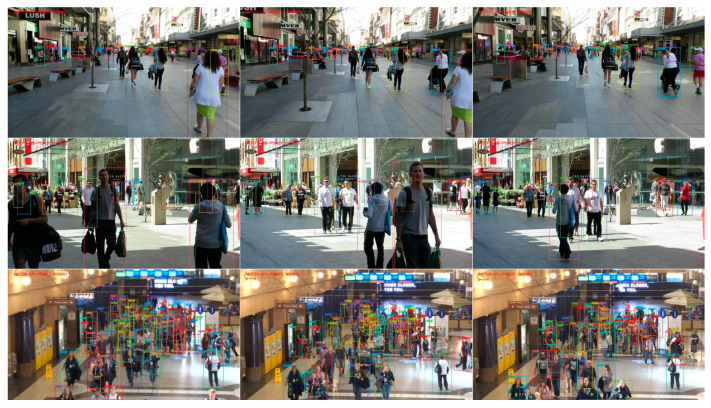
The tracking results for test videos of MOT17-07 (1st row), MOT17-08 (2nd row) and MOT20-08 (3rd row). The targets are marked with bounding boxes of different colors, which represent different identities.

The overall visual results are shown in Figure 7. From the results of MOT17-08 and MOT20-08, we observe that PSMOT manages to detect objects and maintain their identities in challenging scenes where there are frequent occlusions, which is mainly attributed to its abilities to coordinate multiple tasks and exclude interference caused by occlusion. From the results of MOT17-07 and MOT17-08, we also see PSMOT’s robustness against large-scale variations, which is mainly due to the fact that the backbone network aggregates features are from different resolutions.

**Figure 8:**
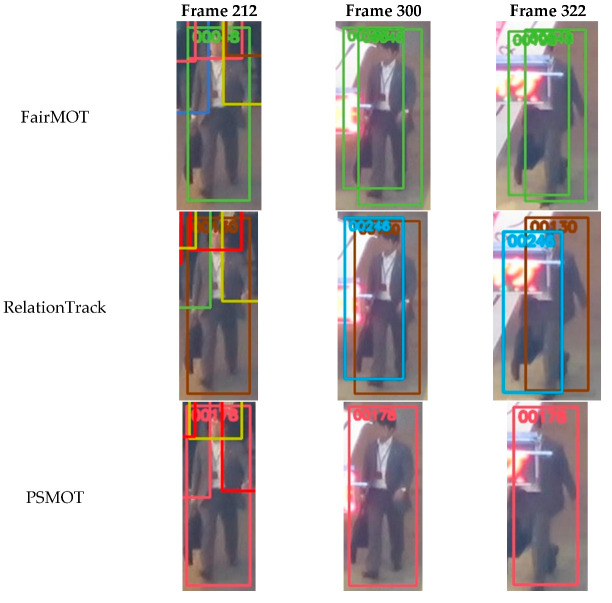
Visual comparison of PSMOT, FairMOT and RelationTrack for handling the false objects on MOT17-03.

Additionally, we also present visual comparisons of PSMOT, FairMOT and RelationTrack in some typical cases for MOT17-03. Figure 8 shows the results of the three trackers for the handling the false objects, from which we can see that, during the tracking process, FairMOT generates duplicate bounding boxes with the same ID number, RelationTrack assigns two different ID numbers to the same object, while PSMOT maintains the unique ID number of the object. Both FairMOT and RelationTrack detect objects and extract their ReID features in a one-shot manner and subsequently utilize NMS to filter out duplicates and objects with low confidence. Because of the fixed thresholds, NMS is unable to filter out the invalid objects completely and accurately, which results in the first and second rows in the figure. In contrast, PSMOT achieves object detection and feature extraction in a two-stage approach: in the first stage, multiple region proposals are generated and, in the second stage, the information within each region is utilized to determine whether the proposal is the background or object. Thus, the two-stage approach, together with the subsequent NMS, eliminates false proposals more efficiently, resulting in the last row in the figure.

**Figure 9:**
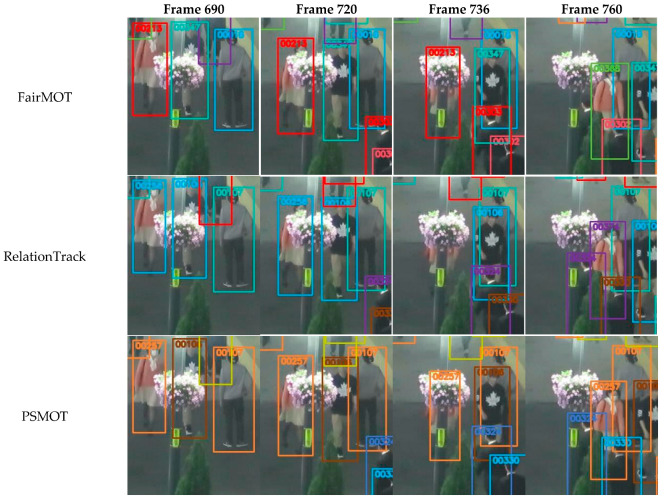
Visual comparison of PSMOT, FairMOT and RelationTrack for handling occlusion on MOT17-03. The lady in the pink blouse is reaching the foreground and becomes partially occluded at frame 690 and 720, is fully in the blind spot at frame 736 and reappears at frame 760.

Figure 9 illustrates the results of the above trackers for handling interference caused by occlusion. It is observed that, at frame 736, since the object is almost completely occluded, none of the methods can detect it, and instead they rely on the motion model to predict the object’s location. FairMOT and PSMOT successfully predict the location of the occluded object using Kalman Filter, while the trajectory-filling strategy employed by RelationTrack falsely filters out this prediction.

Furthermore, at frame 760, both FairMOT and RelationTrack assigns incorrect ID number to the reappearing object, which is attributed to the contamination of the template feature of the corresponding target in the tracking pool—as the object enters the occluded area, its ReID feature is interfered with by occlusion and is directly used to update the template feature of the target in the tracking pool corresponding to the ID number; after the object leaves the occluded area, its newly extracted ReID feature fails to match the contaminated template feature of the original target, thus the object would be recognized as a newcomer or as another target. In contrast, through the object’s classification map with position sensitivity, PSMOT determines the occluded parts of the object and excludes the interference of the occluded parts during the aggregation of the ReID feature, so as to prevent subsequent contamination. As shown in the last row in the figure, PSMOT maintains the correct ID number for the object when it passes through the occluded area.

## 5. Conclusions

In this paper, we unleash the potential of two-stage JDE models for handling feature misalignment and occlusion in MOT. To achieve an ideal two-stage JDE model, efforts are made as follows. To maintain timeliness, the proposed model is fully convolutional, and its RoI-wise process only involves simple statistical operations. Furthermore, the dense and predefined anchoring scheme is replaced with a sparse and adaptive anchoring scheme in the first-stage RPN, which is able to produce more high-quality proposals with fewer anchors; to reach high performance by addressing the multi-task learning problem, feature aggregation and feature disentanglement are accomplished by the model’s encoder–decoder backbone, with a deep level of multi-layer feature fusion and hard parameters sharing, respectively; to improve robustness, position sensitivity is further applied to evaluate the visibility of each proposal’s parts and guide the aggregation of the tasks’ results, while excluding interference. To make a sustainable MOT system, the hierarchical association algorithm in MOTDT is employed. The experimental results exhibits the high performance of the proposed method.

While this study provides valuable insights into the design of the JDE models, there are still some limitations. First, PSMOT is currently implemented only for tracking pedestrians; the experimental results are not comprehensive enough. In our future work, we aim to extend PSMOT to handle scenarios with multiple objects of multiple categories, such as mixed traffic scenarios involving pedestrians, vehicles and bicycles. Second, in this paper we disentangle the shared feature map using multiple non-linear convolutions, which are independent of each other. In principle, this arrangement is hard parameter sharing, which comes at the cost of increased parameter size and computational burden. In the future, we plan to explore networks based on soft parameters sharing to further improve the efficiency of the JDE models.

## Figures and Tables

**Table 1:** Comparison of different backbones based on ResNet-34 on the validation set of the MOT17. The optimal results are shown in bold. The symbol ↑ (↓) indicates that the higher (lower) the value of the metric, the better the performance.

Backbone	MOTA ↑	IDF1 ↑	AssA ↑	DetA ↑	LocA ↑	IDs ↓	Frag ↓
ResNet-34	69.6	70.4	57.8	56.2	78.7	303	631
FPN-34	71.4	72.8	61.1	59.8	81.7	237	370
DLA-34 (ours)	**75.1**	**75.8**	**62.2**	**62.5**	**83.8**	**167**	**269**

**Table 2:** Comparison of different strategies for feature disentanglement on the validation set of the MOT17. The optimal results are shown in bold. The symbol ↑ (↓) indicates that the higher (lower) the value of the metric, the better the performance.

Module	MOTA ↑	IDF1 ↑	AssA ↑	DetA ↑	LocA ↑	IDs ↓	Frag ↓
General	72.9	73.8	59.6	60.1	81.3	224	332
Ours	**75.1**	**75.8**	**62.2**	**62.5**	**83.8**	**167**	**269**

**Table 3:** Comparison of the different strategies for the RPN on the validation set of the MOT17. The optimal results are shown in bold. The symbol ↑ (↓) indicates that the higher (lower) the value of the metric, the better the performance.

Module	Number	MOTA ↑	IDF1 ↑	AssA ↑	DetA ↑	LocA ↑	FPS ↑
Vanilla RPN	300	72.1	72.5	58.5	59.1	79.3	14.3
	1000	73.5	73.9	60.1	60.3	80.6	5.1
GA-RPN (ours)	300	73.2	74.4	59.5	60.2	82.3	**22.4**
	500	75.1	75.8	62.2	62.5	83.8	16.6
	1000	**75.6**	**76.5**	**63.5**	**64.1**	**85.3**	7.4

**Table 4:** Effects of the position sensitivity adopted to handle occlusion on the validation set of the MOT17. The optimal results are shown in bold. The symbol ↑ (↓) indicates that the higher (lower) the value of the metric, the better the performance.

K × K	MOTA ↑	IDF1 ↑	IDs ↓	Frag ↓	FPS ↑
1 × 1	64.9	63	535	878	**20.3**
3 × 3	74.6	75	252	504	18.6
5 × 5	75.1	75.8	167	269	16.6
7 × 7	**75.4**	76.4	133	196	10.3
9 × 9	**75.4**	**76.6**	**122**	**183**	4.1

**Table 5:** Evaluation of the impact of the association scheme on PSMOT. The symbol ↑ (↓) indicates that the higher (lower) the value of the metric, the better the performance. The symbol ✓ means that the corresponding component is employed.

ReID	IoU and Kalman	Hierarchy	MOTA ↑	IDF1 ↑	IDs ↓	Frag ↓	FPS ↑
✓	✓	✓	75.1	75.8	167	269	16.6
✓	✓		74.0	73.6	217	326	19.0
✓			72.7	72.6	279	446	20.3

**Table 6:** Configurations of different versions of PSMOT.

Versions	Max Proposals	Dims of PS Maps
PSMOT-Fast	300	3
PSMOT-Balance	500	5
PSMOT-Pro	1000	7

**Table 7:** Comparisons with typical online MOT methods on the test sets of MOT17. The ‘Params’ in the last column in the table show the parameter size of each model. The symbol ↑(↓) indicates that the higher (lower) the value of the metric, the better the performance.

Tracker	MOTA ↑	IDF1 ↑	AssA ↑	DetA ↑	LocA ↑	IDs ↓	Frag ↓	FPS ↑	Params ↓
FairMOT [6]	73.3	72.3	58.0	60.9	83.6	3303	8073	25.9	24.4 M
RelationTrack [8]	73.8	74.7	61.5	60.6	83.4	1374	2166	8.5	22.7 M
PSMOT-Fast	73.9	74.1	59.1	61.6	83.5	3052	4883	20.0	24.8 M
PSMOT-Balance	75.1	75.8	62.2	62.5	83.8	1896	2804	16.6	24.9 M
PSMOT-Pro	75.9	76.3	63.9	64.3	84.4	1309	2114	6.2	25.0 M

**Table 8:** Comparison of PSMOT with other methods on the test sets of MOT17 and MOT20. The optimal results are shown in red bold and the sub-optimal are in bold and underlined. The symbol ↑ (↓) indicates that the higher (lower) the value of the metric, the better the performance.

Dataset	Tracker	Time	Arch	MOTA ↑	IDF1 ↑	AssA ↑	DetA ↑	LocA ↑	IDs ↓	Frag ↓	FPS ↑
MOT17	TPM [54]	2020	SDE	54.2	52.6	40.9	42.5	80.0	1824	2472	0.8
	TrajE [55]	2021	SDE	67.4	61.2	46.6	53.5	81.5	4019	6613	1.4
	FairMOT [6]	2020	JDE	73.3	72.3	58.0	60.9	83.6	3303	8073	**25.9**
	CSTrack [7]	2020	JDE	74.9	72.6	57.9	61.1	83.3	3567	7668	15.8
	Semi-TCL [56]	2021	JDE	73.3	73.2	59.4	60.4	83.7	2790	8010	--
	RelationTrack [8]	2021	JDE	73.8	74.7	61.5	60.6	83.4	**1374**	**2166**	8.5
	OUTrack [9]	2022	JDE	73.5	70.2	56.7	61.1	**83.8**	4122	7500	**25.9**
	Swin_JDE [10]	2023	JDE	72.3	70.7	57.4	58.5	83.0	2679	3903	4.5
	TubeTK [57]	2020	JDT	63.0	58.6	45.1	51.4	81.1	4137	5727	3.0
	CenterTrack [58]	2020	JDT	67.8	64.7	51.0	53.8	81.5	3039	6102	3.8
	TransCenter [59]	2021	JDT	73.2	62.2	49.7	60.1	83.5	4614	9519	1.0
	TrackFormer [60]	2022	JDT	74.1	68	54.1	60.9	82.8	2829	4221	5.7
	Decode_MOT [61]	2023	JDT	73.2	72	58.9	60.5	83.6	3363	6051	1.8
	MeMOTR [62]	2023	JDT	72.8	71.5	58.4	59.6	83.0	1902	4770	**29.6**
	PSMOT-Fast	---	JDE	73.9	74.1	59.1	61.6	83.5	3052	4883	20.0
	PSMOT-Balance			**75.1**	**75.8**	**62.2**	**62.5**	**83.8**	1896	2804	16.6
	PSMOT-Pro			**75.9**	**76.3**	**63.9**	**64.3**	**84.4**	**1309**	**2114**	6.2
MOT20	FairMOT [6]	2020	JDE	61.8	67.3	54.7	54.7	81.1	5243	7874	13.2
	CSTrack [7]	2020	JDE	66.6	68.6	54.0	54.2	81.5	3196	7632	4.5
	Semi-TCL [10]	2021	JDE	65.2	70.1	56.3	54.6	81.2	4139	8508	**22.4**
	RelationTrack [8]	2021	JDE	67.2	**70.5**	**56.4**	56.8	81.8	4243	8236	4.3
	OUTrack [9]	2022	JDE	**68.6**	69.4	55.6	**57.0**	82.5	2223	5683	12.4
	LMOT [11]	2022	JDE	59.1	61.1	46.8	48.4	83.1	**1398**	2446	**22.4**
	RetinaMOT [8]	2023	JDE	66.8	67.5	53.8	54.5	82.0	1739	3217	22.4
	Swin_JDE [10]	2023	JDE	70.4	69.5	54.8	56.9	82.1	2026	3165	4.1
	MLT [63]	2020	JDT	48.9	54.6	44.1	42.7	80.6	2187	3067	3.7
	TransTrack [64]	2020	JDT	65.0	59.4	45.2	53.3	82.8	3608	11,352	14.9
	TransCenter [59]	2021	JDT	58.5	49.6	37.0	51.7	81.1	4695	9581	1.0
	GSDT [65]	2021	JDT	67.1	67.5	52.7	54.7	81.7	3230	9878	1.5
	TrackFormer [60]	2022	JDT	**68.6**	65.7	53.0	56.7	**83.7**	1532	2474	5.7
	TicrossNet [66]	2023	JDT	60.6	59.3	44.9	51.9	81.4	4266	6969	**31.0**
	Decode_MOT [61]	2023	JDT	67.2	69.0	54.6	54.5	81.4	2805	7084	12.2
	PSMOT-Fast	---	JDE	66.6	68.2	55.2	55.1	81.2	2295	3442	13.2
	PSMOT-Balance			68.2	70.4	56.3	56.3	82.3	1426	**2339**	10.1
	PSMOT-Pro			**69.2**	**72.0**	**58.3**	**58.4**	**83.4**	**1210**	**2032**	3.8

## Data Availability

The data that support the findings of this study are available from the corresponding author upon reasonable request.

## References

[1] Teng S., Hu X., Deng P., Li B., Li Y., Yang D., Ai Y., Li L., Zhe X., Zhu F. (2023). Motion Planning for Autonomous Driving: The State of the Art and Future Perspectives. IEEE Trans. Intell. Veh..

[2] Varghese E.B., Thampi S.M. (2023). A Comprehensive Review of Crowd Behavior and Social Group Analysis Techniques in Smart Surveillance. Intelligent Image and Video Analytics.

[3] Wu H., Nie J., Zhang Z., He Z., Gao M. (2023). Deep Learning-based Visual Multiple Object Tracking: A Review. Comput. Sci..

[4] Voigtlaender P., Krause M., Osep A., Luiten J., Sekar B.B.G., Geiger A., Leibe B. Mots: Multi-object tracking and segmentation. Proceedings of the 2019 IEEE/CVF Conference on Computer Vision and Pattern Recognition.

[5] Wang Z., Zheng L., Liu Y., Li Y., Wang S. Towards real-time multi-object tracking. Proceedings of the Computer Vision-ECCV2020: European Conference.

[6] Zhang Y., Wang C., Wang X., Zeng W., Liu W. (2021). FairMOT: On the Fairness of Detection and Re-identification in Multiple Object Tracking. Int. J. Comput. Vis..

[7] Liang C., Zhang Z., Lu Y., Li B., Zhu S., Hu W. (2020). Rethinking the competition between detection and ReID in Multi-Object Tracking. arXiv.

[8] Yu E., Li Z., Han S., Wang H. (2021). RelationTrack: Relation-aware Multiple Object Tracking with Decoupled Representation. arXiv.

[9] Liu Q., Chen D., Chu Q., Yuan L., Liu B., Zhang L., Yu N. (2022). Online Multi-Object Tracking with Unsupervised Re-IDentification Learning and Occlusion Estimation. Neurocomputing.

[10] Tsai C.Y., Shen G.Y., Nisar H. (2023). Swin-JDE: Joint Detection and Embedding Multi-Object Tracking Based on Swin-Transformer. Eng. Appl. Artif. Intell..

[11] Mostafa R., Baraka H., Bayoumi A. (2022). LMOT: Efficient Light-Weight Detection and Tracking in Crowds. IEEE Access.

[12] Cao J., Zhang J., Li B., Gao L., Zhang J. (2023). RetinaMOT: Rethinking anchor-free YOLOv5 for online multiple object tracking. Complex Intell. Syst..

[13] Dai J., Li Y., He K., Sun J. (2016). R-FCN: Object Detection via Region-based Fully Convolutional Networks. arXiv.

[14] Wang J., Kai C., Shuo Y., Loy C.C., Lin D. (2019). Region Proposal by Guided Anchoring. arXiv.

[15] Yu F., Wang D., Shelhamer E., Darrell T. Deep Layer Aggregation. Proceedings of the 2018 IEEE/CVF Conference on Computer Vision and Pattern Recognition.

[16] Dai J., He K., Li Y., Ren S., Sun J. (2016). Instance-sensitive Fully Convolutional Networks. arXiv.

[17] Chen L., Ai H., Zhuang Z., Shang C. Real-Time Multiple People Tracking with Deeply Learned Candidate Selection and Person Re-Identification. Proceedings of the 2018 IEEE International Conference on Multimedia and Expo (ICME).

[18] Bewley A., Ge Z., Ott L., Ramos F., Upcroft B. (2016). Simple Online and Realtime Tracking. arXiv.

[19] Kalman R.E. (1960). A New Approach to Linear Filtering and Prediction Problems. J. Basic Eng..

[20] Ren S., He K., Girshick R., Sun J. (2015). Faster R-CNN: Towards Real-Time Object Detection with Region Proposal Networks. arXiv.

[21] Yu F., Li W., Li Q., Liu Y., Shi X., Yan J. (2016). POI: Multiple Object Tracking with High Performance Detection and Appearance Feature. arXiv.

[22] Szegedy C., Liu W., Jia Y., Sermanet P., Reed S., Anguelov D., Erhan D., Vanhoucke V., Rabinovich A. (2014). Going Deeper with Convolutions. arXiv.

[23] Lee S., Kim E. (2019). Multiple Object Tracking via Feature Pyramid Siamese Networks. IEEE Access.

[24] Wojke N., Bewley A., Paulus D. Simple online and realtime tracking with a deep association metric. Proceedings of the 2017 IEEE International Conference on Image Processing (ICIP).

[25] Li Z., Cai S., Wang X., Shao H., Niu L., Xue N. Multiple Object Tracking with GRU Association and Kalman Prediction. Proceedings of the 2021 International Joint Conference on Neural Networks (IJCNN).

[26] Sener O., Koltun V. (2018). Multi-Task Learning as Multi-Objective Optimization. arXiv.

[27] Cipolla R., Gal Y., Kendall A. Multi-task Learning Using Uncertainty to Weigh Losses for Scene Geometry and Semantics. Proceedings of the 2018 IEEE/CVF Conference on Computer Vision and Pattern Recognition.

[28] Jocher G. (2022). YOLOv5 Release V6.1. https://github.com/ultralytics/yolov5/releases/tag/v6.1.

[29] Hu W., Li X., Luo W., Zhang X., Maybank S., Zhang Z. (2012). Single and Multiple Object Tracking Using Log-Euclidean Riemannian Subspace and Block-Division Appearance Model. IEEE Trans. Pattern Anal. Mach. Intell..

[30] Izadinia H., Saleemi I., Li W., Shah M. (MP)^2^T: Multiple People Multiple Parts Tracker. Proceedings of the Computer Vision-ECCV2020: European Conference.

[31] Shu G., Dehghan A., Oreifej O., Hand E., Shah M. Part-based multiple-person tracking with partial occlusion handling. Proceedings of the 2012 IEEE/CVF Conference on Computer Vision and Pattern Recognition.

[32] Tang S., Andriluka M., Schiele B. (2014). Detection and Tracking of Occluded People. Int. J. Comput. Vis..

[33] Wu B., Nevatia R. (2007). Detection and Tracking of Multiple, Partially Occluded Humans by Bayesian Combination of Edgelet based Part Detectors. Int. J. Comput. Vis..

[34] Chu Q., Ouyang W., Li H., Wang X., Liu B., Yu N. (2017). Online Multi-Object Tracking Using CNN-based Single Object Tracker with Spatial-Temporal Attention Mechanism. arXiv.

[35] Ess A., Leibe B., Schindler K., Van Gool L. A mobile vision system for robust multi-person tracking. Proceedings of the 2008 IEEE/CVF Conference on Computer Vision and Pattern Recognition.

[36] Zhang S., Benenson R., Schiele B. CityPersons: A Diverse Dataset for Pedestrian Detection. Proceedings of the 2017 IEEE/CVF Conference on Computer Vision and Pattern Recognition.

[37] Zhang S., Xie Y., Wan J., Xia H., Li S.Z., Guo G. (2020). WiderPerson: A Diverse Dataset for Dense Pedestrian Detection in the Wild. IEEE Trans. Multimed..

[38] Dollar P., Wojek C., Schiele B., Perona P. Pedestrian detection: A benchmark. Proceedings of the 2009 IEEE/CVF Conference on Computer Vision and Pattern Recognition.

[39] Milan A., Leal-Taixe L., Reid I., Roth S., Schindler K. (2016). MOT16: A benchmark for multi-object tracking. arXiv.

[40] Xiao T., Li S., Wang B., Lin L., Wang X. (2016). Joint Detection and Identification Feature Learning for Person Search. arXiv.

[41] Zheng L., Zhang H., Sun S., Chandraker M., Yang Y., Tian Q. Person Re-identification in the Wild. Proceedings of the 2017 IEEE/CVF Conference on Computer Vision and Pattern Recognition.

[42] Dave A., Khurana T., Tokmakov P., Schmid C., Ramanan D. (2020). TAO: A Large-Scale Benchmark for Tracking Any Object. arXiv.

[43] Dendorfer P., Rezatofighi H., Milan A., Shi J., Cremers D., Reid I., Roth S., Schindler K., Leal-Taixé L. (2020). MOT20: A benchmark for multi object tracking in crowded scenes. arXiv.

[44] Luiten J., Osep A., Dendorfer P., Torr P., Geiger A., Leal-Taixe L., Leibe B. (2021). HOTA: A Higher Order Metric for Evaluating Multi-object Tracking. Int. J. Comput. Vis..

[45] Li Y., Huang C., Nevatia R. Learning to associate: HybridBoosted multi-target tracker for crowded scene. Proceedings of the 2009 IEEE Conference on Computer Vision and Pattern Recognition.

[46] Bernardin K., Stiefelhagen R. (2008). Evaluating Multiple Object Tracking Performance: The CLEAR MOT Metrics. EURASIP J. Image Video Process..

[47] Ristani E., Solera F., Zou S., Cucchiara R., Tomasi C. (2016). Performance Measures and a Data Set for Multi-Target, Multi-Camera Tracking. arXiv.

[48] Lin T.Y., Maire M., Belongie S., Bourdev L., Girshick R., Hays J., Perona P., Ramanan D., Zitnick C.L., Dollár P. (2014). Microsoft COCO: Common Objects in Context. arXiv.

[49] Kingma D.P., Ba J. (2014). Adam: A Method for Stochastic Optimization. arXiv.

[50] Shrivastava A., Gupta A., Girshick R. Training Region-Based Object Detectors with Online Hard Example Mining. Proceedings of the 2016 IEEE/CVF Conference on Computer Vision and Pattern Recognition.

[51] Dvornik N., Mairal J., Schmid C. (2018). Modeling Visual Context is Key to Augmenting Object Detection Datasets. arXiv.

[52] He K., Zhang X., Ren S., Sun J. Deep Residual Learning for Image Recognition. Proceedings of the 2016 IEEE/CVF Conference on Computer Vision and Pattern Recognition.

[53] Lin T.Y., Dollár P., Girshick R., He K., Hariharan B., Belongie S. Feature Pyramid Networks for Object Detection. Proceedings of the 2017 IEEE/CVF Conference on Computer Vision and Pattern Recognition.

[54] Peng J., Wang T., Lin W., Wang J., See J., Wen S., Ding E. (2020). TPM: Multiple Object Tracking with Tracklet-Plane Matching. Pattern Recognit..

[55] Girbau A., Giró-i-Nieto X., Rius I., Marqués F. (2021). Multiple Object Tracking with Mixture Density Networks for Trajectory Estimation. arXiv.

[56] Li W., Xiong Y., Yang S., Xu M., Wang Y., Xia W. (2021). Semi-TCL: Semi-Supervised Track Contrastive Representation Learning. arXiv.

[57] Pang B., Li Y., Zhang Y., Li M., Lu C. TubeTK: Adopting Tubes to Track Multi-Object in a One-Step Training Model. Proceedings of the 2020 IEEE/CVF Conference on Computer Vision and Pattern Recognition.

[58] Zhou X., Koltun V. (2020). Tracking Objects as Points. arXiv.

[59] Xu Y., Ban Y., Delorme G., Gan C., Rus D., Alameda-Pineda X. (2023). TransCenter: Transformers with Dense Representations for Multiple-Object Tracking. IEEE Trans. Pattern Anal. Mach. Intell..

[60] Meinhardt T., Kirillov A., Leal-Taixe L., Feichtenhofer C. TrackFormer: Multi-Object Tracking with Transformers. Proceedings of the 2022 IEEE/CVF Conference on Computer Vision and Pattern Recognition.

[61] Lee S.H., Park D.H., Bae S.H. (2023). Decode-MOT: How Can We Hurdle Frames to Go Beyond Tracking-by-Detection?. IEEE Trans. Image Processs..

[62] Gao R., Wang L. (2023). MeMOTR: Long-Term Memory-Augmented Transformer for Multi-Object Tracking. arXiv.

[63] Zhang Y., Sheng H., Wu Y., Wang S., Ke W., Xiong Z. (2020). Multiplex Labeling Graph for Near-Online Tracking in Crowded Scenes. IEEE Internet Things J..

[64] Sun P., Cao J., Jiang Y., Zhang R., Xie E., Yuan Z., Wang C., Luo P. (2020). TransTrack: Multiple Object Tracking with Transformer. arXiv.

[65] Wang Y., Kitani K., Weng X. (2020). Joint Object Detection and Multi-Object Tracking with Graph Neural Networks. arXiv.

[66] Fukui H., Miyagawa T., Morishita Y. (2023). Multi-Object Tracking as Attention Mechanism. arXiv.

